# Improvement of Health-Related Quality of Life After Roux-en-Y Gastric Bypass Related to Weight Loss

**DOI:** 10.1007/s11695-016-2468-6

**Published:** 2016-11-28

**Authors:** Valerie M. Monpellier, Evangelia E. Antoniou, Edo O. Aarts, Ignace M. C. Janssen, Anita T. M. Jansen

**Affiliations:** 1 Nederlandse Obesitas Kliniek (Dutch Obesity Clinic), Obesity International, Huis ter Heide, postbus 601, 3700 AP Zeist, the Netherlands; 2 Faculty of Psychology and Neuroscience, Maastricht University, Maastricht, the Netherlands; 3 Department of Surgery, Rijnstate Hospital, Arnhem, the Netherlands

**Keywords:** Health-related quality of life, Roux-en-Y gastric bypass, Weight loss, Impact of weight on quality of life-lite, RAND-36

## Abstract

**Introduction:**

Effect of bariatric surgery on health-related quality of life (HRQOL) varies greatly. This might be caused by the diversity in questionnaires used to assess HRQOL and the weight loss of the studied population. This study assesses the relationship between weight loss and HRQOL in primary Roux-en-Y gastric bypass (RYGB) patients by using an obesity-specific (impact of weight on quality of life-lite, IWQOL-lite) and a generic (RAND-36) questionnaire.

**Methods:**

HRQOL and weight parameters were assessed before and 15 and 24 months after RYGB surgery. HRQOL was assessed by using IWQOL-lite (an obesity-specific questionnaire consisting of one total score and five domains) and RAND-36 (a generic questionnaire consisting of two subtotal scores, the physical health summary (PHS) and mental health summary (MHS), and nine scales).

**Results:**

Two thousand one hundred thirty-seven patients were included. HRQOL improved significantly after RYGB. Preoperative BMI was negatively related to baseline PHS (*p* < 0.001) and IWQOL-lite total (*p* < 0.001). Percentage total weight loss (%TWL) was positively related to HRQOL score at both follow-up moments. Change in HRQOL from baseline to 24 months was related to %TWL at 24 months in both subtotals of RAND-36 and IWQOL-lite total score (*p ≤* 0.001 in all).

**Conclusion:**

HRQOL improves after RYGB. Higher %TWL is related to greater improvement in HRQOL and better HRQOL 15 and 24 months after RYGB. The variance in the effect of RYGB surgery on HRQOL can be explained by the questionnaire used and weight loss of the population.

## Introduction

Health-related quality of life (HRQOL) is currently considered a vital parameter after medical treatment worldwide [1]. In obese patients, HRQOL is significantly impaired; therefore, HRQOL improvement is one of the primary outcome measurements after bariatric surgery [2–5]. Common belief is that bariatric surgery positively affects HRQOL; however, recent publications question this positive effect [5–8]. These publications show great variance in the effect of bariatric surgery and address two possible causes for this. First, HRQOL is assessed with numerous questionnaires, since there is no specific questionnaire to assess HRQOL in bariatric surgery patients [5, 6]. Second, weight loss might also influence HRQOL [7, 8].

HRQOL questionnaires can be divided in two groups: obesity-specific questionnaires, like impact of weight on quality of life-lite (IWQOL-lite), and general questionnaires, like the RAND-36. In the obese population, body weight was the main determinant of improvement of HRQOL when IWQOL-lite was used; outcome of a general questionnaire was only partially dependent on body weight [2, 9, 10].

Strikingly, the relationship between weight loss and HRQOL has been studied only sparsely in the bariatric population, and with conflicting results [8, 11, 12]. Even fewer studies have assessed the effect of bariatric surgery with a general and an obesity-specific questionnaire [8, 13].

In total, HRQOL is an important outcome after bariatric surgery. However, there is still a knowledge gap regarding the effect of bariatric surgery on HRQOL and the influence of weight loss on HRQOL. This study evaluated HRQOL before and after primary laparoscopic Roux-en-Y gastric bypass (RYGB) by using the IWQOL-lite and the RAND-36. Secondly, the relationship between HRQOL and weight loss was assessed.

## Methods

### Patient Selection and Standard Treatment

This is a retrospective analysis of prospectively collected data; data was collected up to July 2015. Patients were selected from the database of the Nederlandse Obesitas Kliniek (Dutch Obesity Clinic part of Obesity International), the largest, outpatient clinic for treatment of bariatric patients in the Netherlands which provides the structured care for over 50% of the Dutch bariatric population. All patients were screened according to IFSO-criteria. In addition to bariatric surgery, the treatment program consists of pre- and post-operative group counseling by a multidisciplinary team (dietician, a psychologist, a physical therapist, and a medical doctor).

Since 2012, HRQOL was routinely assessed in the treatment program, starting with the RAND-36 and later also adding IWQOL-lite. This treatment program was enrolled over the different clinics at several time points during 2012 and 2013. The questionnaires were administered at preoperative screening and at 15 and 24 months after surgery.

### Inclusion Criteria

All 2562 patients who had undergone a primary RYGB before 2014 were selected from the database. Patients were included when IWQOL-lite or RAND-36 results were available before surgery and at least one time post-surgery.

### Health-Related Quality of Life

#### IWQOL-Lite

The IWQOL-lite is a 31-item questionnaire which assesses the impact of weight on quality of life in five domains. This questionnaire has shown good validity and reliability in obese patients (test-rest reliability *r* = 0.74–0.91; internal consistency, Cronbach’s alpha 0.85) and was used before in the bariatric population [5, 14]. In addition to a total score, there are scores on five scales: physical function, self-esteem, sexual life, public distress, and work [15].

#### RAND-36

The RAND-36 is a general HRQOL questionnaire with 36 questions and 9 scales: emotional role functioning, social functioning, vitality, physical functioning, mental health, bodily pain, general health perceptions, health change, and physical role functioning. From these scales, two subtotals can be calculated: physical health summary (PHS) and mental health summary (MHS) [16]. The RAND-36 has also been validated for the obese population (test-rest reliability *r* = 0.94; internal consistency, Cronbach’s alpha 0.96) [16].

For both questionnaires, a high score represents a higher HRQOL and scores range from 0 to 100. The smallest difference in score, which the patient perceives as beneficial, is the minimal clinically important difference (MCID). In previous research with bariatric patients, the MCID threshold for RAND-36 was 5, and for IWQOL-lite the threshold was 12 [17].

### Body Weight and Other Parameters

Body weight was assessed at the same time points as HRQOL; height was assessed during preoperative screening. Weight loss was calculated and reported as stated in the most recent guidelines: body mass index (BMI in kg/m^2^), BMI difference (ΔBMI), and percent total weight loss (% TWL) [5]. The following parameters were also registered: gender, age, and comorbidities (diabetes mellitus, hypertension, obstructive sleep apnea syndrome, hypercholesterolemia, and osteoarthritis) at baseline.

### Statistical Analysis

Descriptive statistics summarized the patients’ characteristics. Differences from baseline to follow-up points in HRQOL were analyzed with repeated measures ANOVA. Differences from 15 to 24 follow-up points in HRQOL were analyzed with paired sample *T* tests. A regression analysis was performed to examine the relationship between BMI at baseline (BL), 15 months (15 M), and 24 months (24 M), and HRQOL total scores. Linear regression was also performed to study the relationship between weight change (%TWL) and HRQOL scores at 15 and at 24 months follow-up. In addition, change in HRQOL (ΔHRQOL) from baseline to both follow-up moments, 15 and 24 months, was calculated. And two new variables were created, ΔHRQOL15 and ΔHRQOL24:

$$ \begin{array}{l}\varDelta \mathrm{HRQOL}15=\frac{15\mathrm{month}\kern0.4pc  HRQOL\kern0.28em \mathrm{score}-\mathrm{baseline}\kern0.28em  HRQOL\kern0.28em \mathrm{score}}{\mathrm{maximum}\kern0.28em  HRQOL\kern0.28em \mathrm{score}}\\ {}\varDelta \mathrm{HRQOL}24=\frac{24\mathrm{month}\kern0.28em  HRQOL\kern0.28em \mathrm{score}-\mathrm{baseline}\kern0.28em  HRQOL\kern0.28em \mathrm{score}}{\mathrm{maximum}\kern0.28em  HRQOL\kern0.28em \mathrm{score}}\end{array} $$

The relationship of ΔHRQOL and %TWL at both follow-up moments was first examined with a bivariate analysis for total scores. Secondly, a multiple regression was performed adjusting for baseline BMI, gender, age, and comorbidities (diabetes mellitus, hypertension, obstructive sleep apnea syndrome, hypercholesterolemia, and osteoarthritis).

All statistical analysis was performed using the SPSS (version 23) statistical software. In all analysis, a *p* value <0.05 was considered statistically significant.

## Results

### Study Population

The study population consisted of 2137 patients; 425 patients were excluded because of insufficient data. All patients had a follow-up longer than 15 months; in 44 patients (2.1%) no weight loss data was available at 15 months. Follow-up was longer than 24 months in 1411 patients; in 23.5% (*n* = 332) no weight loss data were available.

Preoperative RAND-36 scores were available in 2133 patients, at 15 months for 2074 patients, and at 24 months for 1036 patients. For IWQOL-lite, results of 2130 patients were available preoperatively; at 15 months there were 1953 results and at 24 months 612 results.

Mean age was 45.8 years and 82.5% of the population was female (see Table 1). Mean baseline BMI was 44.5 kg/m^2^ (SD ± 5.8). At 15 months follow-up, mean BMI was 30.7 kg/m^2^ (SD ± 5.1) and TWL was 31.0%; at 24 months, mean BMI was 30.7 kg/m^2^ (SD ± 5.2) and mean TWL was 31.1%.

**Table 1:** Demographics of included population at baseline (*n* = 2137), 15 months (*n* = 2093), and 24 months (*n* = 1079)

	Mean ± SD	Percentage (no.)
Age, years	45.8 ± 10.7	
Follow-up, months	26.8 ± 5.9	
Female gender		82.5% (1762)
Diabetes mellitus		23.5% (503)
Hypertension		39.2% (838)
Obstructive sleep apnea syndrome		11.1% (237)
Hypercholesterolemia		20.1% (429)
Osteoarthritis		12.8% (274)
No comorbidity		43.3% (925)
BL BMI, kg/m^2^	44.5 ± 5.8	
15 M BMI, kg/m^2^	30.7 ± 5.1	
24 M BMI, kg/m^2^	30.7 ± 5.2	
15 M ΔBMI, kg/m^2^	13.8 ± 4.1	
24 M ΔBMI, kg/m^2^	13.9 ± 4.3	
15 M %TWL	31.0 ± 7.9	
24 M %TWL	31.1 ± 8.4	

*BL* baseline, *15 M* 15 months follow-up, *24 M* 24 months follow-up, *BMI* body mass index, *ΔBMI* change in BMI, *%TWL* % total weight loss

### HRQOL

All total scores and subscales of RAND-36 significantly improved in both when comparing BL with 15 months and BL with 24 months (*p <* 0.001 in all, see Table 2). RAND-36 scores of all scales at 15 months were significantly higher (*p* < 0.05 in all) than at 24 months. IWQOL-lite scores also improved significantly in all scales, when comparing BL with both follow-up moments (*p <* 0.001 in all, see Table 2). IWQOL-lite scores at 24 months follow-up were lower than at 15 M; this difference was not statistically significant.

**Table 2:** RAND-36 and IWQOL-lite scores at baseline and at 15 and 24 months follow-up

RAND-36	BL (*n* = 2137)		15 M (*n* = 2074)		24 M (*n* = 1036)	
	Mean	±SD	Mean	±SD	Mean	±SD
Subtotals						
Physical health summary	52.2	22.2	80.3*	19.0	78.6*†	20.7
Mental health summary	65.8	19.3	78.1*	19.1	75.3*†	20.0
Scales						
Emotional role functioning	76.9	37.3	86.6*	30.6	83.2*†	33.9
Social functioning	68.4	25.6	83.3*	23.1	80.8*†	23.7
Vitality	48.2	18.5	65.4*	20.1	62.5*†	20.5
Physical functioning	51.9	24.5	86.9*	20.1	85.4*°	21.7
Mental health	69.7	17.0	77.0*	18.4	74.5*†	18.3
Bodily pain	59.5	26.2	78.3*	25.2	77.2*°	26.2
General health perceptions	42.7	18.8	71.2*	19.5	69.3*†	20.3
Health change	35.4	24.9	92.7*	18.6	76.2*†	26.9
Physical role functioning	54.7	41.7	85.1*	30.8	82.6*†	32.6
IWQOL-lite	BL (*n* = 2130)		15 M (*n* = 1953)		24 M (*n* = 612)	
	Mean	±SD	Mean	±SD	Mean	±SD
Total	56.9	18.7	91.1*	12.5	89.9*	12.7
Scales						
Physical function	42.6	21.3	89.8*	13.7	88.7*	13.1
Self-esteem	48.3	26.3	87.5*	18.3	85.7*	19.0
Sexual life	60.0	31.6	88.8*	20.2	86.8*	22.3
Public distress	62.0	24.1	94.2*	12.8	93.6*	12.7
Work	71.3	22.4	94.9*	12.1	94.7*	12.0

*BL* baseline, *15 M* 15 months follow-up, *24 M* 24 months follow-up

*Significant difference compared to baseline, *p* < 0.001

†Significant difference compared to 15 months follow-up, *p* ≤ 0.001

°Significant difference compared to 15 months follow-up, *p* ≤ 0.05

### Baseline BMI and HRQOL

A higher baseline BMI was significantly associated with a lower baseline PHS score (*R* ^2^ = 0.005, F(1,2131) = 10.539, *p* < 0.001). There was no significant association between baseline BMI and baseline MHS score. IWQOL-lite total score was also negatively associated with BMI (*R* ^2^ = 0.037, F(1,2128) = 82.420, *p* < 0.001).

### Follow-up Weight and HRQOL

PHS score was significantly negatively related to BMI at 15 months (*R* ^*2*^ = 0.013, F(1,2056) = 26.355, *p* < 0.001) and 24 months (*R* ^2^ = 0.005, F(1,1019) = 5.403, *p =* 0.020). MHS was only associated with BMI at 24 months (*R* ^2^ = 0.004, F(1,2056) = 3.956, *p* = 0.047); this was also negative. IWQOL-lite total score was negatively associated with BMI at both 15 months (*R* ^2^ = 0.094, F(1,1935) = 201.653, *p* < 0.001) and 24 months (*R* ^2^ = 0.139, F(1601) = 97.412, *p* < 0.001). A higher BMI was associated with a lower HRQOL in both RAND-36 subtotals and the IWQOL-lite total score.

Fifteen and 24 months scores of PHS, MHS, and IWQOL-lite total score were significantly associated with %TWL (*p ≤* 0.001 in all). A higher %TWL was associated with a higher HRQOL in all RAND-36 subtotals and the IWQOL-lite total score.

### Weight Loss and Change in HRQOL

At 15 months, ΔHRQOL ranged from 0.07 to 0.57 for RAND-36 scores, and ΔHRQOL15 ranged from 0.23 to 0.47 for IWQOL-lite scores. Significant correlations with %TWL were found in the physical functioning scale (*r*_s_(2054) = 0.096, *p* < 0.001) and general health perception scale (*r*_s_(2054) = 0.091, *p* < 0.001) of RAND-36. For the IWQOL-lite, all scales had significant correlations; the highest correlation was with self-esteem scale (*r*_*s*_(1931) = 0.147, *p* < 0.001).

ΔHRQOL15 of PHS and IWQOL-lite total score were significantly associated with %TWL (*p* < 0.001 in both). Also after adjusting for baseline BMI, gender, age, and comorbidities (diabetes mellitus, hypertension, obstructive sleep apnea syndrome, hypercholesterolemia, and osteoarthritis), %TWL was still significantly related to ΔHRQOL15 for PHS and IWQOL-lite total score (see Table 3). For ΔHRQOL15 of MHS, there was no significant association (*p* = 0.213).

**Table 3:** Linear regression analysis for ΔHRQOL based on %TWL at both follow-up moments

	β*	SE	95% CI	*p* value	*R* ^2^
15 months					
RAND-36 PHS	0.002	0.001	0.001–0.004	<0.001	0.024
RAND-36 MHS	0.001	0.001	0.000–0.002	0.169	0.004
IWQOL-lite total	0.003	0.001	0.002–0.004	<0.001	0.055
24 months					
RAND-36 PHS	0.003	0.001	0.002–0.005	<0.001	0.038
RAND-36 MHS	0.002	0.001	0.001–0.004	0.003	0.026
IWQOL-lite total	0.004	0.001	0.003–0.006	<0.001	0.079

*ΔHRQOL* change in HRQOL from baseline to 15 and 24 months follow-up, *TWL* total weight loss, *PHS* physical health summary, *MHS* mental health summary

*Adjusted for baseline BMI, gender, age, and comorbidities (diabetes mellitus, hypertension, obstructive sleep apnea syndrome, hypercholesterolemia, and osteoarthritis)

ΔHRQOL24 ranged from 0.05 to 0.42 for RAND-36 scores and from 0.24 to 0.47 for IWQOL-lite scores.

At 24 months, the highest correlation with %TWL was found in the physical functioning scale (*r*_s_(1034) = 0.128, *p* < 0.001) and general health perception scale (*r*_s_(1034) = 0.124, *p* < 0.001) of RAND-36. In the IWQOL-lite, the highest correlation was with self-esteem scale (*r*_s_(609) = 0.230, *p* = 0.104).

ΔHRQOL24 of PHS, MHS, and IWQOL-lite total score was significantly associated with %TWL (*p* < 0.001 in all). After adjusting for baseline BMI, gender, age, and comorbidities (diabetes mellitus, hypertension, obstructive sleep apnea syndrome, hypercholesterolemia, and osteoarthritis), %TWL remained significantly related to ΔHRQOL24 for all total scores (see Table 3).

## Discussion

The purpose of this study was to evaluate the effect of primary RYGB on HRQOL and assess the relationship between weight loss and HRQOL with two HRQOL questionnaires. Our results show that HRQOL significantly improves after primary RYGB when assessed with both a generic (RAND-36) and an obesity-specific (IWQOL-lite) questionnaire. A higher BMI is associated with a lower HRQOL before and after surgery, while more weight loss is associated with higher improvement of HRQOL and better HRQOL score at 15 and 24 months follow-up. The correlations between weight (loss) and HRQOL were higher with the IWQOL-lite compared to the RAND-3.

The effect of bariatric surgery on HRQOL was questioned in recent literature [6, 8]. This study shows that HRQOL significantly improves after RYGB. After 15 months follow-up, there was a statistically significant decline for all RAND-36 scores. This effect was also observed up to 5 years post-surgery in the SOS study [18]. However, the reduction was only clinically relevant for one scale, the health change scale. The mean score of this scale at 24 months was still higher than the mean score of the normal population sample [19]. For the IWQOL-lite, there was also a slight decline, but none of the differences were statistically significant or clinically relevant. It seems that HRQOL stabilized 15 months post-surgery. This might be explained by the fact that patients experience an enormous improvement in HRQOL in the first 15 months. But after 12–15 months, in most patients, weight stabilizes and thereby the additional changes that the patients experience in the months afterwards seem only small. Moreover, the HRQOL scores at 15 months are higher than the normal population scores; maybe there is no room left for further improvement.

Patients with a lower BMI before and after RYGB surgery generally had a better HRQOL. Total IWQOL-lite was related to presurgical BMI and BMI at both follow-up moments. In the obese population, it was shown before that BMI was more related to the IWQOL-lite scores compared to RAND-36 scores [9, 10]. This study is the first to show that this also applies in the pre- and post-bariatric population.

For the generic questionnaire, BMI was associated with physical health. This is in concordance with more studies assessing RAND-36 scores in post-bariatric patients [8]. It is likely that the physical effects of morbid obesity are substantial and thereby also influence general HRQOL. Mental health of the RAND-36 was only negatively associated with BMI at 24 months post-surgery.

Patients with a higher weight loss had a better HRQOL; total scores of RAND-36 and IWQOL-lite were all significantly positively associated with %TWL. The effect of weight loss on HRQOL has been evaluated in several studies with various questionnaires; however, the expected outcome had not been as clear as in our study [11, 12, 20–23]. In addition, TWL was 31.1% at 24 months in our population, which might explain the greater association of weight loss and HRQOL. Other studies had much smaller populations and generally used percentage excess weight loss as a weight loss parameter. The higher number of patients in our study and the use of %TWL might have influenced the results.

Our results show that the variation in effect of bariatric surgery on HRQOL which was described recently can at least in part be explained by the diversity in questionnaires used to assess HRQOL [6]. Change in HRQOL was the highest in the IWQOL-lite scales, ranging from 23 to 47%. For RAND-36, mean change ranged from 5 to 57%. The correlations between %TWL and ΔHRQOL were also the highest in the IWQOL-lite scales at 15 and 24 months follow-up. And RAND-36 MHS was not significantly associated with ΔHRQOL at 15 months. To avoid influence of baseline HRQOL on outcome, we calculated a ΔHRQOL.

In our study population maximum weight loss was achieved at an average of 15 months; therefore, we chose to use 15 and 24 months to evaluate HRQOL. The large sample size enabled us to identify even small effects of weight loss on HRQOL. In previous research, there was deterioration in HRQOL seen, which was explained by weight regain [18]. However, due to the strict follow-up protocol, our study population had undergone no weight regain (yet); weight was still declining or remained stable in all patients at 24 months. Looking at previous research, it is very interesting to further investigate HRQOL and identify other factors which could influence the stabilization of HRQOL.

Only RYGB patients were included in this analysis to ensure accurate data analysis without the bias of difference in weight loss. Because of the increase in the number of patients receiving other types of surgery, such as gastric sleeve, future research should focus on the effect of gastric sleeve surgery on HRQOL.

Because the studied population was the first population in which HRQOL was systematically assessed, not all patients completed HRQOL at all follow-up moments. Despite this, the included population is still the largest bariatric population in which two types of HRQOL questionnaires were assessed.

## Conclusion

Reported variance in the effect of RYGB on HRQOL can be explained by both the questionnaire used and the weight loss of the researched population; this should be taken into account when the effect of bariatric surgery on HRQOL is studied. HRQOL of prebariatric patients is low, and even lower in patients with a higher BMI. HRQOL improves significantly after RYGB when measured with both a specific and a generic questionnaire. However, the improvement in HRQOL is higher when an obesity-specific questionnaire is used. The positive effect on HRQOL is greater in patients with a lower BMI and higher %TWL up to 24 months after RYGB. Thus, more weight loss not only has a beneficial effect on medical comorbidities but also positively influences patient’s well-being.

For future studies reporting HRQOL after surgery, mean weight loss and preoperative score should be taken into account.
